# Key events in cancer: Dysregulation of SREBPs

**DOI:** 10.3389/fphar.2023.1130747

**Published:** 2023-03-08

**Authors:** Yunkuo Li, Shouwang Wu, Xiaodong Zhao, Shiming Hao, Faping Li, Yuxiong Wang, Bin Liu, Difei Zhang, Yishu Wang, Honglan Zhou

**Affiliations:** ^1^ Department of Urology, The First Hospital of Jilin University, Changchun, China; ^2^ Key Laboratory of Pathobiology, Ministry of Education, Jilin University, Changchun, China

**Keywords:** SREBPs, lipid metabolism, fatty acids, cholesterol, cancer therapy

## Abstract

Lipid metabolism reprogramming is an important hallmark of tumor progression. Cancer cells require high levels of lipid synthesis and uptake not only to support their continued replication, invasion, metastasis, and survival but also to participate in the formation of biological membranes and signaling molecules. Sterol regulatory element binding proteins (SREBPs) are core transcription factors that control lipid metabolism and the expression of important genes for lipid synthesis and uptake. A growing number of studies have shown that SREBPs are significantly upregulated in human cancers and serve as intermediaries providing a mechanistic link between lipid metabolism reprogramming and malignancy. Different subcellular localizations, including endoplasmic reticulum, Golgi, and nucleus, play an indispensable role in regulating the cleavage maturation and activity of SREBPs. In this review, we focus on the relationship between aberrant regulation of SREBPs activity in three organelles and tumor progression. Because blocking the regulation of lipid synthesis by SREBPs has gradually become an important part of tumor therapy, this review also summarizes and analyzes several current mainstream strategies.

## 1 Introduction

Lipids, also known as fats, are classified into two types: lipoids (such as phospholipids, glycolipids, and sterols) and fats (such as triglycerides and sterols). Sterols mainly include cholesterol, sex hormones, and vitamin D (Cheng et al., 2018; Long et al., 2018). Lipids are widely distributed in cellular organelles and serve as important building blocks of all membranes. Additionally, lipids play a critical role as energy sources, signaling molecules, and secondary messengers (Snaebjornsson et al., 2020; Matsushita et al., 2021). As the availability of nutrients consistently changes with tumor progression, cancer cells in the tumor microenvironment use lipid metabolism to support their rapid proliferation, survival, migration, invasion, and metastasis (Bian et al., 2021).

**FIGURE 1:**
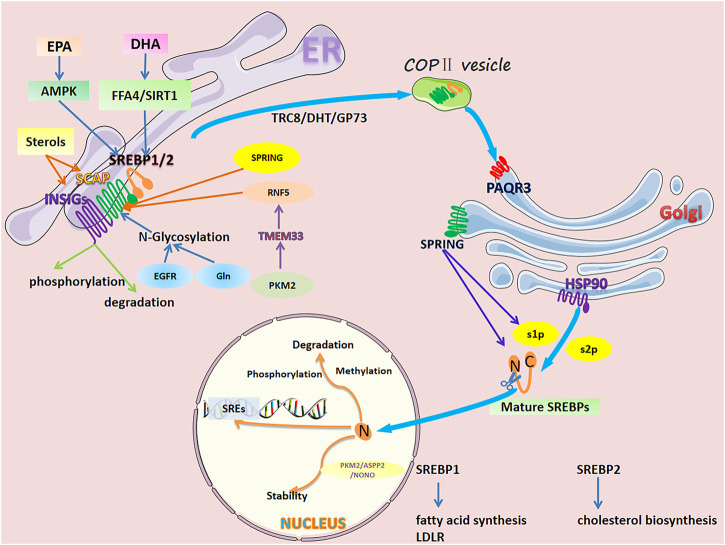
Regulation of SREBP1/2 in cancer cells. The activation process of SREBPs is as follows. Inactive SREBPs reside in the ER membrane and interact with SCAP. The N-terminal domain of SCAP combines with INSIG, forming an INSIG/SCAP/SREBP complex anchored to the ER. When sterol levels decrease, SCAP dissociates from INSIGs and mediates SREBPs into COPII vesicles, transporting the SCAP/SREBP complex from the ER to the golgi. In the golgi, SREBPs are sequentially cleaved by S1P and S2P, releasing their transcriptionally active N-terminal domains. After cleavage, mature SREBPs translocate to the nucleus and bind to SREs and E-boxes within target gene promoters. However, SREBPs are delicately and complexly regulated in individual organelles. In the ER, sterol levels directly affect the dissociation of SCAP from INSIGs. Long-chain polyunsaturated fatty acids (DHA and EPA) inhibit SREBPs at the mRNA and protein levels. N-glycosylation of SCAP, RNF5-induced degradation, SPRING-induced reduction, phosphorylation, and degradation of INSIGs all affect the transport of SREBPs to the golgi. In the golgi, PAQR3 promotes SCAP/SREBP localization and enhances the processing of SREBPs. HSP90 binds the SREBP-SCAP complex, stabilizing it and facilitating its transport from the ER to the golgi. SPRING, a necessary cofactor for the cleavage of SREBPs, directly affects the level of SREBP. In the nucleus, mature SREBPs undergo phosphorylation, methylation, and ubiquitination-related degradation. Additionally, protein-protein interactions affect their stability.

Lipogenesis and lipid uptake is transcriptionally controlled by sterol regulatory element binding proteins (SREBPs) (Horton et al., 2002). There are two SREBP proteins in humans: SREBP1 encoded by the *SREBF1* gene and SREBP2 encoded by the *SREBF2* gene (Brown and Goldstein, 1997; Osborne and Espenshade, 2009). SREBP1 has two isoforms: SREBP1a and SREBP1c, produced through the use of alternative transcription start sites and the difference in the first exon (exon 1a and exon 1c) (Eberlé et al., 2004) that mainly regulate genes controlling fatty acid synthesis (Brown and Goldstein, 1997; Horton et al., 2002; Horton et al., 2003a; Osborne and Espenshade, 2009). SREBP2 regulates cholesterol biosynthesis gene expression (Brown and Goldstein, 1997; Horton et al., 2003a; Horton et al., 2003b). Inactive SREBPs reside in the endoplasmic reticulum (ER) membrane and interact with SREBP cleavage-activating protein (SCAP), a polytopic transmembrane protein (Gong et al., 2015) (Figure 1). The N-terminal domain of SCAP can combine with the insulin-induced gene protein (INSIG), forming an INSIG/SCAP/SREBP complex anchored to the ER (Yabe et al., 2002; Yang et al., 2002). When sterol levels decrease, SCAP dissociates from INSIG and mediates SREBPs into the Coat Protein complex II (COPII) vesicles, transporting the SCAP/SREBP complex from the ER to the Golgi. In the Golgi, SREBPs are sequentially cleaved by site 1 and 2 proteases (S1P and S2P), releasing their transcriptionally active N-terminal domains. After cleavage, mature SREBPs translocate to the nucleus and bind to SREs and E-boxes within target gene promoters (Nohturfft et al., 2000; Sun et al., 2007).

Cancer cells require high levels of lipid synthesis and uptake to support their continued replication. Highly expressed SREBPs play an important role in lipid reprogramming in a variety of cancers, including gastric cancer (Sun et al., 2020), colon cancer (Gao et al., 2019), breast cancer (Bao et al., 2016), glioblastoma (Han et al., 2020), prostate cancer (Ettinger et al., 2004; Huang et al., 2012), hepatocellular carcinoma (Yahagi et al., 2005; Li et al., 2014a; Heo et al., 2020), and thyroid cancer (Li et al., 2020; Huang et al., 2022). The activity of SREBPs is regulated by different mechanisms at different subcellular localizations, including the ER, Golgi, and nucleus. In this review, these three organelles serve as the main thread throughout the entire process of SREBP maturation and activity. In each organelle, we discuss the regulation of SREBPs by tumor cells through various signaling pathways, which further regulate tumor cell lipid uptake, lipid production (fatty acids (FAs) and cholesterol), and lipolysis to serve the tumor cells. Because blocking the activity of SREBPs has gradually become an important measure for cancer treatment, we summarize and analyze several current mainstream strategies at the end of the review.

## 2 Regulation of SREBPs in the ER

Normally, SREBPs are anchored to the ER in the form of an INSIG/SCAP/SREBP complex. SREBPs must undergo the following two stages to function: dissociation of the SCAP/SREBPs complex from INSIG in the ER and subsequent translocation to the Golgi. We reviewed many related studies on the regulation of SREBPs in the ER and found that four main factors affected these two stages: 1) classical regulation of sterols, 2) regulation of long-chain fatty acids, 3) dependent and independent mTOR signaling pathways, and 4) stability of INSIG/SCAP/SREBP complexes.

### 2.1 Regulation of sterols

Sterol fluctuations in the ER regulate SREBP activation (Figure 1). Decreased sterol levels facilitate the dissociation of SCAP from INSIGs and incorporation of SCAP/SREBP complexes into COPII-coated vesicles (Menendez and Lupu, 2007). Cholesterol disrupts the interaction between SCAP and COPII by binding to SCAP and retains SREBPs in the ER (Shimano and Sato, 2017). Cholesterol loading reduced the expression of SCAP and the translocation of SREBP1 to the nucleolus, as well as the expression of key rate-limiting enzymes (fatty acid synthase (FASN) and acetyl-CoA carboxylase 1 (ACC1)) in *de novo* fatty acid synthesis, inhibiting hepatocellular carcinoma (HCC) progression *in vivo* and *in vitro* (Zhao et al., 2019). 25-hydroxycholesterol (25-HC), an oxidized cholesterol, retains SREBPs in the ER stronger than cholesterol (Adams et al., 2004; Eberlé et al., 2004). Cancer cells are sensitive to sterols, and the expression of cholesterol and fatty acid biosynthesis genes (*SREBF1/2*, stearoyl-CoA desaturase (*SCD*), *FASN*) was inhibited by 25-HC in cancer cells, such as glioma, breast cancer, and prostate cancer cells (Williams et al., 2013). Similarly, 25-HC acts as an inhibitor of SREBPs and reduces hepatitis C Virus (HCV) replication in hepatoma cells. 25-HC and its synthesizing enzyme cholesterol 25-hydroxylase also inhibit HCV infection by inhibiting the maturation of SREBPs (Xiang et al., 2015). Changes in cholesterol transport and esterification can affect the activation of SREBPs and the occurrence and development of tumors. p53 can induce the transcription of cholesterol transporter ATP-binding cassette transporter A1 (ABCA1). Loss of p53 or ABCA1 ablation inhibited the retrograde transport of cholesterol from the plasma membrane to the ER, thereby promoting the maturation of SREBP2 and hepatocellular carcinoma in mice (Moon et al., 2019). ER-resident sterol o-acyltransferase (SOAT) reduces ER cholesterol levels by esterifying cholesterol to form cholesteryl esters and sequestering it into lipid droplets (Chang et al., 2006; Walther and Farese, 2009). Inhibition of SOAT resulted in ER cholesterol accumulation and decreased cholesterol esterification, thereby inhibiting SREBP1-regulated gene expression, glioblastoma growth, and prostate cancer cell invasion (Yue et al., 2014; Geng et al., 2016; Navarro-Imaz et al., 2019).

### 2.2 Regulation of long-chain fatty acids

Long-chain fatty acids characterized by a double bond on the third carbon atom (the hydroxycarboxylic acid chain counted from the methyl end) are called omega-3 polyunsaturated fatty acids (Calder, 2018). Omega-3 polyunsaturated fatty acids can inhibit SREBP1c in two ways: inhibition of nuclear abundance of SREBP1c and proteasome-mediated degradation of SREBP1c (Botolin et al., 2006; Scorletti and Byrne, 2013; Gnoni and Giudetti, 2016). Eicosapentaenoic acid [EPA; 20: 5(omega-3)] and docosahexaenoic acid [DHA; 22: 6(omega-3)] are ultra-long-chain highly unsaturated omega-3 fatty acids (Figure 1). Interestingly, DHA and EPA play an important role in inhibiting the proteolytic activation of SREBPs through an inhibitory mechanism distinct from sterols in cancer. In human breast cancer MCF-7 cells, DHA inhibits pAKT signaling, thereby inhibiting the precursor of SREBP1and its mature form expression and cancer cell proliferation (Huang et al., 2017). In liver cancer cells, DHA inhibits *SREBP1c* at the mRNA and protein levels; however, the inhibition of SREBP1c expression by DHA is related to free fatty acid receptor 4 (FFA4, a G protein-coupled receptor and target of DHA (Hirasawa et al., 2005)), and its inhibitory effect is attenuated by FFA4 knockdown (Kang et al., 2018). DHA protects against colon carcinogenesis by inhibiting insulin-induced activation of SREBP1 and cyclooxygenase-2 expression by upregulating SIRT1 (Song et al., 2014). DHA activation can activate SREBP2 in SW620 colon cancer cells. However, activated SREBP2 induces only a few target genes (low-density lipoprotein receptor (*LDLR*) and the first specific enzyme in cholesterol biosynthesis, *SQS/FDFT1*), and cholesterol biosynthesis remains reduced (Størvold et al., 2009). EPA, an agent that improves lipid metabolism (Carpentier et al., 2006), inhibits the development of steatohepatitis and HCC in Pten-deficient mice by increasing AMPKα1 and PPARα expression and decreasing SREBP1c expression (Ishii et al., 2009). In a human hepatoma cell line (HepG2), oxidized EPA inhibited the expression of SREBP1c and its downstream target genes more effectively than EPA (Nanthirudjanar et al., 2013). In addition to the regulatory effects of sterols and fatty acids, both ethanol and androgen can play a role in regulating SREBPs in cancer cells (Swinnen et al., 1997; You et al., 2002).

### 2.3 mTOR-dependent and mTOR-independent signaling pathways

**FIGURE 2:**
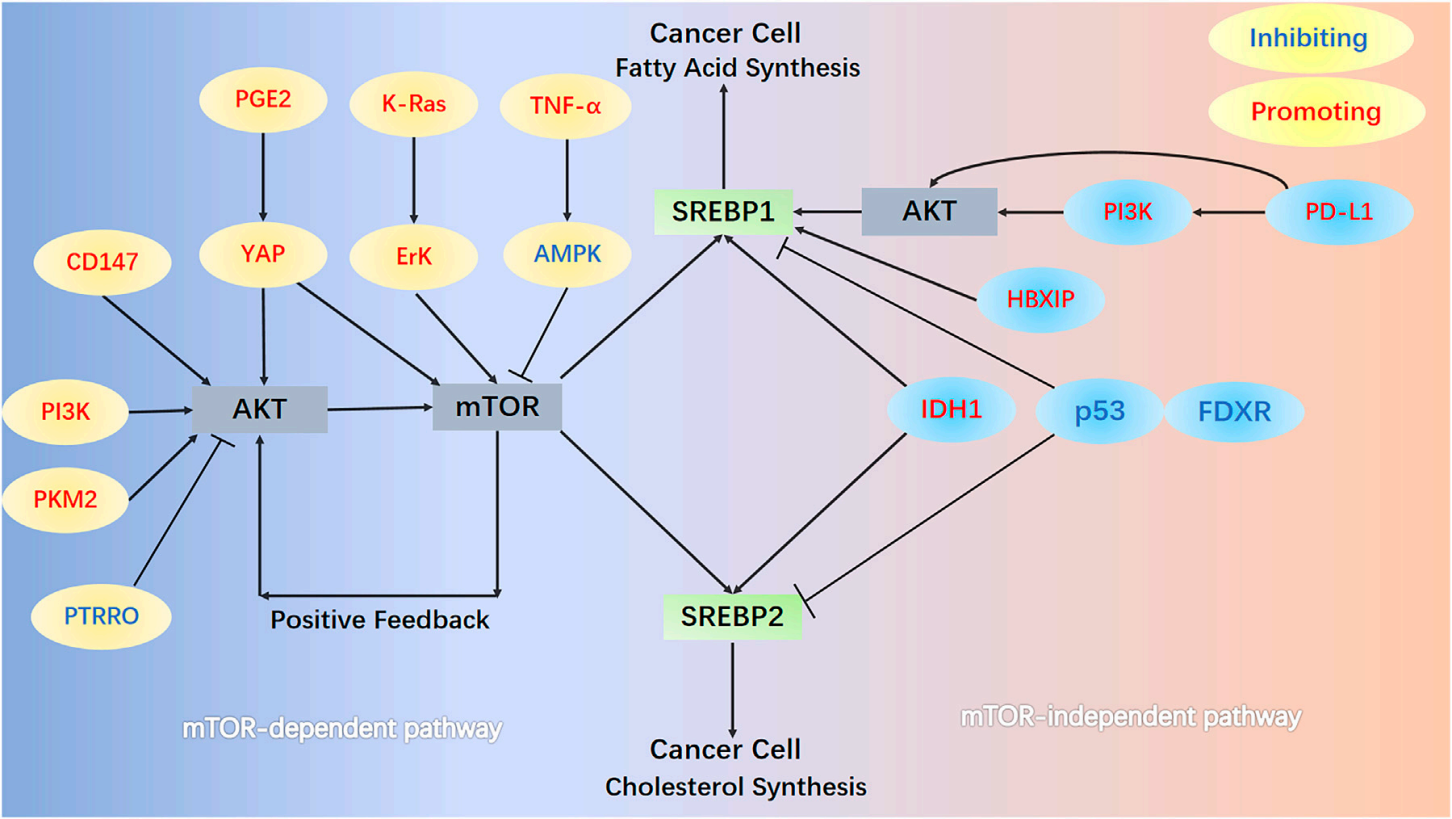
mTOR-dependent and mTOR-independent signaling pathways. In the mTOR-dependent signaling pathways, AKT/mTOR/SREBPs activate SREBPs in cancer cells. Numerous protein molecules can directly or indirectly (through AKT) act on mTOR, thereby regulating the activation of SREBPs. In the PI3K/AKT/mTOR/SREBP1 pathway, mTOR further enhances AKT signaling in melanoma cells through positive feedback. In the mTOR-independent pathway, HBXIP and PD-L1 act directly on SREBP1 and promote its activation. Mutation of the R132H site of IDH1, loss of p53, and FDXR all promote the activation of SREBP1 and SREBP2.

Multiple signaling pathways, classified into mTOR-dependent or mTOR-independent mechanisms, can regulate the activation of SREBPs in a lipid-independent manner in cancer (Figure 2). The most studied is the PI3K/AKT/mTOR/SREBP1 signaling pathway, which is often abnormally activated in tumor cells. In human melanoma cells, ganglioside GD3, expressed as a melanoma antigen, regulates the activity of SREBPs and cholesterol biosynthesis through the PI3K-AKT-mTORC1 signaling pathway. Interestingly, the presence of positive feedback to this signaling pathway through PI3K-AKT-mTORC1-enhanced SREBPs signaling further boosts Akt signaling in GD3-expressing human melanoma cells (Yamauchi et al., 2011). A new study revealed a novel mechanism of the PI3K/AKT/mTOR/SREBP1 signaling pathway that protects cancer cells by inhibiting ferroptosis (an iron-dependent form of cell death caused by the accumulation of phospholipid peroxides). Persistent activation of the PI3K/AKT/mTOR/SREBP1 signaling pathway mediates adipogenesis and renders cancer cells resistant to ferroptosis in PI3K-mutant breast cancer mice. SREBP1 inhibits ferroptosis in cancer cells by upregulating its transcriptional target SCD1 and producing monounsaturated fatty acids (Yi et al., 2020). Pyruvate kinase M2 (PKM2) is expressed at high levels in most cancers and catalyzes the last rate-limiting step in glycolysis. Downregulation of PKM2 reduces FASN expression and inhibits bladder cancer cell growth by significantly reducing the phosphorylation of both AKT and mTOR and inactivating the AKT/mTOR/SREBP1c signaling pathway (Tao et al., 2019). High expression of CD147, a transmembrane glycoprotein, is closely related to tumor growth, invasion, and angiogenesis (Su et al., 2009; Voigt et al., 2009). In HCC cells, the AKT/mTOR signaling pathway, activated by CD147, upregulates the expression of SREBP1c and its target genes *FASN* and *ACC* and promotes fatty acid synthesis. Concurrently, CD147 also inhibits fatty acid oxidation by inhibiting the fatty acid oxidation signaling pathways of p38 MAPK/PPARα/CPT1A and ACOX1, thereby reprogramming lipid metabolism and increasing cancer cell invasiveness (Li et al., 2015a). Protein tyrosine phosphatase receptor type O (PTPRO) suppresses tumors tumorigenesis and progression in several cancers. PTPRO, which has the opposite effect but a similar mechanism to CD147, inhibits the occurrence and metastasis of CRC by regulating two signaling pathways: AKT/mTOR/SREBP1/ACC1 and MAPK/PPARα/ACOX1 (Dai et al., 2022). The tumor microenvironment (TME) is considered a key factor in tumor progression and interaction with cancer cells (Quail and Joyce, 2013; Shi et al., 2017). Mesenchymal stem cells, an important component of the TME, increase the expression of cyclooxygenase 2 under hypoxic conditions, thereby increasing the secretion of prostaglandin E_2_ (PGE_2_). Hippo signaling pathway effector Yes-associated protein 1 (YAP), activated by PGE2, promotes hepatocellular carcinoma progression by upregulating the AKT/mTOR/SREBP1 signaling pathway (Liu et al., 2019). Interestingly, in non-tumorigenic MCF10A epithelial cells, YAP activates mTORC1/SREBP1 *via* serum and glucocorticoid-regulated kinase 1, rather than the AKT-mediated mTORC1/SREBP1 mechanism, which conflicts with the performance of hepatocellular carcinoma (Vaidyanathan et al., 2022). K-Ras activates the mTORC1/SREBPs (SREBP1 and SREBP2) signaling pathway and enhances the autonomous growth of breast cancer cells by activating Erk with minimal activation of Akt (Ricoult et al., 2016). Tumor necrosis factor-alpha inhibits the key regulator of energy homeostasis AMP-activated protein kinase (AMPK) and its downstream pathway mTOR/SREBP1, inducing lipid accumulation in human hepatoma HepG2 cells (Lv et al., 2015).

Cancer cells can also activate SREBP and support their increased lipid requirements for growth through an mTOR-independent mechanism. The relationship between upregulation of programmed death 1 ligand 1 (PD-L1) expression and epithelial-mesenchymal transition (EMT) plays a key role in the progression of multiple cancers (Alsuliman et al., 2015; Qiu et al., 2018). PD-L1 can directly induce EMT by upregulating SREBP1c in renal cell carcinoma, promoting cancer cell migration and invasion (Wang et al., 2015). Furthermore, PD-L1 activates SREBP1 *via* the PI3K/AKT signaling pathway, which can promote EMT and invasion of sorafenib-resistant HCC cells (Xu et al., 2020a). The expression of Hepatitis B X-interacting protein (HBXIP) in clinical breast cancer tissues positively correlates with the expression of FASN, contributing to abnormal lipid metabolism and the growth of cancer cells. Oncoprotein HBXIP directly interacts with liver X receptor-α (LXR) to co-activate and upregulate the transcription of SREBP1c and its target gene *FASN* (Zhao et al., 2016). Isocitrate dehydrogenase 1 (IDH1) is frequently mutated in human gliomas, especially the R132H mutation of IDH1 (Yan et al., 2009). IDH1^R132H^ induces shunting of carbon from glycolysis to *de novo* synthesis of lipids and increases expression of *SREBPs* (mRNA levels of *1a*, *1c* and *2*). IDH1^R132H^ is partially mediated by the SREBP1a signaling pathway and promotes glioma cell proliferation, growth, and migration (Zhu et al., 2013). SREBP1, upregulated by IDH1^R132H^, enhances p21 expression (independent of the p53 signaling pathway) and inhibits phosphorylation of retinoblastoma protein, thereby slowing cell cycle progression in glioma cells (Miyata et al., 2013). Ferredoxin reductase (FDXR) and p53 work reciprocally and play key roles in iron homeostasis in tumors (Hwang et al., 2001; Liu and Chen, 2002; Zhang et al., 2017). Deficiency of p53 and FDXR activates SREBP1/2 and leads to increased cellular cholesterol and triglyceride levels by reducing ABCA1 expression. Meanwhile, deficiency of p53 and FDXR predisposes mice to spontaneous tumors, hepatic steatosis, and inflammation (Zhang et al., 2022).

### 2.4 Stability of INSIG/SCAP/SREBP complexes

Sterol and FA fluctuations and mTOR-dependent and -independent signaling pathways can regulate the INSIG/SCAP/SREBP complex in the ER. The N-terminal domain of SCAP can combine with INSIG1/2, forming an INSIG/SCAP/SREBP complex anchored to the ER. The relationship between INSIG/SCAP/SREBP is like that of an anchor, an anchor chain, and a ship. When the stability of INSIG/SCAP in cancer cells is affected, it also affects the “ship” heading to the Golgi for the next step of cutting activation (Figure 1). Under low sterol conditions, SCAP N-glycosylation mediated by glucose at three asparagine (N) positions N263, N590, and N641 *via* the SCAP protein is a prerequisite for SCAP/SREBP transport from the ER to the Golgi (Cheng et al., 2016). N-glycosylation of SCAP reduces its linkage to INSIG-1 and directs the transport of the SCAP/SREBP complex from the ER to the Golgi (Guo, 2016). In glioblastoma, SREBP1, regulated by SCAP N-glycosylation, is highly activated (Guo et al., 2009a; Guo et al., 2009b; Guo et al., 2011; Cheng et al., 2015). EGFR signaling enhances SCAP N-glycosylation and protein levels by promoting glucose uptake, which triggers its dissociation from INSIG1. Dissociation of SCAP induces adipogenesis and glioblastoma growth through activation of SREBP1 (Cheng et al., 2015). Ammonia released from glutamine can also activate glucose-regulated N-glycosylated SCAP and dissociate from INSIG, leading to the translocation and activation of SREBP1, thereby promoting adipogenesis and tumor growth (Cheng et al., 2022). Degradation, reduction, or increase of SCAP affects the translocation and activation of SREBP. ER transmembrane protein 33 (TMEM33), a downstream effector of PKM2 upregulated upon loss of PKM2, regulates the activation of SREBPs. Upregulated TMEM33 recruits an E3 ligase, RNF5, and promotes the degradation of SCAP. Interestingly, depletion of PKM2 reduced breast cancer cell growth; however, systemic PKM2 knockdown accelerated tumor growth in allografts (Liu et al., 2021). SREBP-regulated gene (*SPRING/C12ORF49*), as a glycosylated Golgi-resident membrane protein, plays a decisive role in the SREBPs signaling pathway. In Hap1 and Hepa1-6 hepatoma cells, ablation of SPRING results in a reduction of SCAP and its mislocalization to the Golgi and decreases SREBPs signaling, independent of sterol status (Loregger et al., 2020). TRC8, encoding an E3-ubiquitin ligase and as an ER membrane-associated protein, is a putative tumor suppressor disrupted in a family of hereditary renal cell carcinomas (Gemmill et al., 2002). TRC8 is able to bind SREBP2 and SCAP to form the TRC8-SREBP2-SCAP complex, which blocks the interaction between SCAP and Sec24, one of the COPII proteins responsible for the transport of SREBP2 to the Golgi (Irisawa et al., 2009). The enhancement of SCAP-SREBPs interaction plays an important role in increasing the transport of SREBPs to the Golgi and the activation of SREBPs. High expression of dihydrotestosterone (DHT) or Golgi Protein 73 (GP73) can elevate SCAP-SREBP1 interaction and its trafficking to the Golgi, leading to increased nuclear SREBP1 and subsequent adipogenesis (Yang et al., 2017; Seidu et al., 2021). A more direct and increasingly interesting approach through pharmacological or genetic inhibition of SCAP can significantly inhibit tumor growth in various cancer models (Li et al., 2019; Liu et al., 2020; Lim et al., 2021). Strikingly, a recent study contradicts popular belief that suppressing SREBP by depletion of SCAP in the liver exacerbates liver carcinogenesis. This is due to inhibition of the SCAP/SREBP signaling pathway altering the fatty acid composition of phosphatidylcholine, resulting in ER stress and hepatocyte injury (Kawamura et al., 2022).

Phosphorylation, expression changes, and degradation of INSIGs all alter the translocation of ER-resident SREBPs to the Golgi. In human HCC cells, K-ras mutation and receptor tyrosine kinase activation can phosphorylate cytosolic phosphoenolpyruvate carboxykinase 1 (PCK1, as the gluconeogenesis rate-limiting enzyme) at Ser90 by activating AKT. Translocation of phosphorylated PCK1 to the ER, where it phosphorylates INSIG1 at Ser207 and INSIG2 at Ser151, uses GTP as a phosphate donor on the ER. This phosphorylation, in turn, reduces the binding of INSIGs to sterols, thereby disrupting the interaction between INSIGs and SCAP and releasing the SCAP-SREBP complex for translocation to the Golgi. Ultimately, activation of SREBP proteins (SREBP1 or SREBP2) resulted in the *in vitro* proliferation of HCC cells and carcinogenesis in mice (Xu et al., 2020b). INSIG2 expression can be inhibited by insulin signaling and Akt activation by reducing *INSIG2* mRNA levels (Yecies et al., 2011). In esophageal squamous cell carcinoma, phospholipid biosynthesis/remodeling enzyme lysophosphatidylcholine acyltransferase 1 (LPCAT1) expression is high and positively correlated with SREBP1 expression in the nucleus of tumor tissue. LPCAT1 downregulates INSIG-1 expression by activating EGFR, thereby promoting SREBP1 translocation and cholesterol synthesis (Tao et al., 2021). Excess intracellular cholesterol is esterified by SOAT1 to form lipid droplets (LDs) for storage and to maintain ER cholesterol homeostasis. Inhibition of SOAT1 results in blockage of cholesterol esterification and LDs formation, allowing cholesterol accumulation in the ER. Cholesterol accumulation enhances SCAP and INSIG binding and leads to reduced adipogenesis and tumor suppression (Geng and Guo, 2017).

## 3 Regulation of SREBPs in the golgi

The translocation of the SCAP-SREBP complex from the ER to the Golgi can be triggered by the binding of COPII to SCAP (Figure 1). Membrane-bound S1P and S2P on the Golgi continuously cleave SREBPs and release their transcriptionally active N-terminal domains. Pharmacological inhibition of S1P blocks SREBP2 activation and Golgi complex ATF6 protein cleavage in human hepatoma cells, causing ER stress and contributing to apoptotic cell death (Lebeau et al., 2018). S1P may serve as a novel metabolic target, as its pharmacological inhibition impedes SREBP2 activation and cholesterol synthesis in glioblastoma (Caruana et al., 2017). Interestingly, pharmacological inhibition of S2P also inhibits the intramembrane proteolysis of ATF6 and SREBP1 (but not SREBP2). In castration-resistant prostate cancer and liposarcoma, it may serve as a new therapeutic target (Guan et al., 2011; Guan et al., 2012; Guan et al., 2015). SPRING, a cofactor that controls the maturation of S1P, localizes to the Golgi and is required for the cleavage of its substrates, including SREBPs. SPRING correlates with SREBP-regulated lipid metabolism-related genes. Loss of SPRING reduces mature (cleaved) SREBP levels, inhibits nuclear translocation of SREBPs, and reduces cancer cell proliferation in the absence of cholesterol. SPRING regulates SREBP processing because it interacts with the N-glycosylated form of MBTPS1 to catalyze the proteolytic cleavage of its substrate SREBPs. Notably, in the absence of MBTPS1 activity, the Golgi–ER cycle of SCAP is dysfunctional (Bayraktar et al., 2020; Xiao et al., 2021). Heat shock protein 90 (HSP90) binds the SREBP-SCAP complex, stabilizing it and promoting its transport from the ER to the Golgi. Deletion of HSP90β significantly reduces neutral lipid and cholesterol content by degrading mature SREBPs *via* the Akt-GSK3β-FBW7 signaling pathway (Zheng et al., 2019). Progesterone and fat receptor 3 (PAQR3), a Golgi-anchored membrane protein, plays an important role in tumor suppression by negatively regulating the Raf kinase and AKT signaling pathways (Feng et al., 2007; Xie et al., 2008; Zhang et al., 2010). The anchor protein of SCAP/SREBP in the ER and Golgi is INSIGs and PAQR3, respectively. PAQR3 promotes SCAP/SREBP localization in the Golgi and links it to the Golgi complex, enhancing SREBP processing and increasing cellular cholesterol levels (Xu et al., 2015a).

## 4 Regulation of SREBPs in the nucleus

SREBPs release their transcriptionally active N-terminal domains after cleavage in the Golgi. Mature (cleaved) SREBPs translocate to the nucleus as homodimers, subsequently binding to SREs and E-boxes within the promoters of target genes. In the nucleus, two factors are involved in the regulation of SREBPs and cancer: 1) The transcriptional regulation of SREBPs and 2) the function of SREBPs as transcription factors. Rapid degradation of the ubiquitin-proteasome signaling pathway and multiple chemical modifications (especially phosphorylation and methylation) are the greatest obstacles to nuclear SREBP activity as transcription factors. In addition, microRNAs (miRNAs) are key regulators of metabolism and play an important role in the regulation of SREBPs. Therefore, we summarize the associations of miRNAs, SREBPs, and cancer in a separate section.

### 4.1 Transcriptional control of SREBPs

There are two modes of transcriptional regulation of SREBPs. First, the *SREBF1* and *SREBF2* contain SREs in their promoters; these SREs mediate feed-forward transcriptional regulation (Sato et al., 1996; Amemiya-Kudo et al., 2002). Transcription of the genes encoding SREBP-1c is induced by insulin, which activates its promoter through SREs (Foretz et al., 1999; Dif et al., 2006). Feed-forward regulation of SREBPs also activates the expression of miR-33a and miR-33b encoded within introns of *SREBF1* and *SREBF2* (Brown et al., 2010; Najafi-Shoushtari et al., 2010), thereby suppressing the expression of ABCA1 and reducing efflux of newly synthesized cholesterol (Tall et al., 2008). Second, LXR-α and LXR-β mediate the transcriptional regulation of SREBPs by forming heterodimers with retinoic X receptors (RXR) (Repa et al., 2000). Ectopic overexpression of peroxisome-proliferator-activated receptor-gamma (PPARγ) co-activator-1alpha in the hepatoma line further enhances the abundance of *SREBP1c* mRNA in an LXR/RXR-dependent manner (Oberkofler et al., 2004).

In recent years, new mechanisms have been discovered for the transcriptional control of SREBPs in cancer. The *SREBP1a* promoter (−436 to −398 region) contains binding motifs for transcription factors C/EBP, which belong to a family of basic leucine zipper proteins (Qiao et al., 2013). Recent studies have shown that C/EBP-α and SREBP1 are significantly upregulated in human cancers, expanding a mechanistic link between altered lipid metabolism and malignancy (Guo et al., 2011; Li et al., 2012; Lee et al., 2017; Pang et al., 2021). Hepatitis B virus X protein (HBx) activates SREBP1a transcription *via* C/EBP-α, interacts with LXR-α in HCC cells, and recruits cAMP-response element binding protein (CREB) binding protein to the *SREBP1c* promoter (Na et al., 2009; Qiao et al., 2013). Breast cancer cells secrete several growth factors, including receptor activators for nuclear factor-κB ligand (RANKL), which effectively promote osteoclast formation and activation, leading to excessive bone resorption (Blake et al., 2014; Bellanger et al., 2017). RANKL-induced CREB activation stimulates transcription and activation of SREBP2, which then translocates into the nucleus, promoting breast cancer metastasis and aggravating breast cancer-associated osteolysis (Jie et al., 2019).

### 4.2 Chemical modification and stability

Nuclear SREBPs are rapidly degraded by the ubiquitin-proteasome signaling pathway, suggesting that transcription of their target genes is tightly controlled by nuclear SREBP stability. Therefore, the chemical modification (especially phosphorylation and methylation) and stability of SREBPs in the nucleus are particularly important (Figure 1). Fbw7 interacts with the nuclear form of SREBP1a and phosphorylates it at T426 and S430 dependent on GSK3, resulting in enhanced ubiquitination and degradation (Sundqvist et al., 2005). In mitotic cells, the protein kinase Polo-like kinase 1 phosphorylates threonine residues at the docking site of nuclear SREBP1 with Fbw7, blocking the interaction between SREBP1 and Fbw7 and reducing nuclear SREBP1 Fbw7-dependent degradation (Bengoechea-Alonso and Ericsson, 2016). Protein arginine methyltransferase 5 induces arginine methylation (dimethylation of R321) of SREBP1a, preventing SREBP1a from being phosphorylated by GSK3β at S430 and dissociating from Fbw7, thereby evading degradation by the ubiquitin-proteasome signaling pathway. Methylation-stabilized SREBP1a increases lipid synthesis and accelerates cancer cell growth *in vivo* and *in vitro* (Liu et al., 2016). During mitosis, Cdk1 also mediates S439 phosphorylation of SREBP1, leading to increased stability of mature SREBP1 and supporting lipid synthesis (Bengoechea-Alonso and Ericsson, 2006). PKM2 interacts with nuclear SREBP1a and promotes Thr-59 phosphorylation of SREBP1a, which further enhances nuclear SREBP1a protein stability. Thr-59 phosphorylation of nuclear SREBP1a not only promotes the proliferation of hepatoma cells but also negatively correlates with overall survival in patients with hepatocellular carcinoma (Zhao et al., 2018). Interestingly, AMPK can interact with SREBP1c and SREBP2 and directly phosphorylate them at Ser372. In HepG2 hepatoma cells exposed to high glucose, SREBP1c nuclear translocation and lipid accumulation can be inhibited by Ser372 phosphorylation of SREBP1c (Li et al., 2011). The tumor suppressor ASPP2, as a p53 activator, can directly interact with nuclear SREBP2 and inhibit the transcriptional activity of its target genes, especially key enzymes of the mevalonate signaling pathway, leading to tumor growth in hepatocellular carcinoma (Liang et al., 2019). NONO binds to nuclear SREBP1a *via* residue Y267 and increases nuclear SREBP1a protein stability, thereby stimulating breast cancer cell proliferation and tumor growth *in vitro* and *in vivo* (Zhu et al., 2016). Thus, phosphorylation, methylation, ubiquitination, and protein-protein interactions all regulate the activity of nuclear SREBPs. The activity of nuclear SREBPs can also be regulated by controlling their localization and accumulation. Phosphatidic acid phosphatase LPIN1 promotes nuclear localization of mature SREBP1 by mTORC1-mediated phosphorylation and cytoplasmic retention, which in turn regulates *SREBP1* promoter activity and nuclear SREBP1 protein abundance (Peterson et al., 2011). In human HepG2 hepatoma cells, restriction of phosphatidylcholine (a major component of membranes) biosynthesis promotes nuclear SREBP1 accumulation and increases nuclear localization of SREBP1, leading to lipid droplet formation (Walker et al., 2011). Malic enzyme 2 promotes SREBP1 maturation and nuclear localization by inhibiting AMPK phosphorylation, which promotes preneuronal-mesenchymal transition in glioblastoma (Yang et al., 2021). Interestingly, nuclear accumulation of SREBP1 was blocked by the mTORC1 inhibitor rapamycin (Porstmann et al., 2008).

### 4.3 Regulation of SREBPs by microRNAs

**FIGURE 3:**
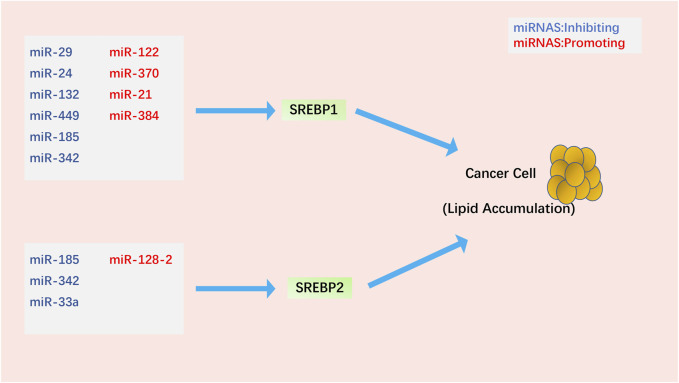
Regulation of SREBPs by miRNAs in cancer cells. Most miRNAs affect lipid accumulation in cancer cells by regulating SREBP1. Notably, miR-185 and miR-342 simultaneously inhibit the activation of SREBP1 and SREBP2.

miRNAs are small non-coding RNAs that are key regulators of metabolism and play an important role in regulating SREBPs in cancer (Figure 3). miR-122, the first miRNA associated with metabolic control, is mainly expressed in the liver (Rottiers and Näär, 2012). miR-122 has a clear and important role in up-regulating SREBPs through the following mechanism: INSIG1 restricts the cholesterol biosynthetic signaling pathway by anchoring the transcription factor SREBPs on the ER and causing degradation of the rate-limiting enzyme HMGCR in cholesterol biosynthesis (Iliopoulos et al., 2010; Shibata et al., 2013; Zhai et al., 2017). miR-122 regulates SREBPs activation by degrading SREBPs’ anchor protein INSIG1, which regulates the expression of LH receptor mRNA binding protein, thereby mediating LH receptor mRNA levels (Menon et al., 2013; Menon et al., 2015; Menon et al., 2018). In Huh7 liver cancer cells, miR-122 regulates the use of polyadenylation sites in *INSIG1* mRNA and inhibits the translation of *INSIG1* isoform mRNA, thereby affecting the activation of SREBPs (Norman et al., 2017). In addition, miR-122 can be controlled by miR-370, further regulating the expression of SREBP1c and Cpt1α, thereby affecting the expression of other genes involved in lipid metabolism in HepG2 liver cancer cells (Iliopoulos et al., 2010). miR-29 inhibits the growth of glioblastoma cells *in vitro* after transfection (Xu et al., 2015b) and correlates with lipid metabolism signaling pathways in hepatoma and liver cells (Kurtz et al., 2014; Xu et al., 2016). EGFR signaling enhances miR-29 expression by upregulating the expression of SCAP/SREBP1, which transcriptionally activates a specific SRE motif in the *miR-29* promoter. Interestingly, miR-29 inversely represses SCAP and SREBP1 expression and drives glioblastoma growth by interacting with the 3′-UTR of SCAP and SREBP1 (Ru et al., 2016; Ru and Guo, 2017). TUT1, a nucleotidyl transferase and regulator of microRNA abundance, upregulates miRNA-24 and miRNA-29 to suppress the expression levels of PPARγ and SREBP1c and lipogenesis in osteosarcoma cells (Zhu et al., 2014).

The molecular link between miRNAs, SREBPs, and SIRT1 (an oncogene closely related to tumorigenesis (Yeung et al., 2004; Kuzmichev et al., 2005; Hida et al., 2007; Huffman et al., 2007)) in cancer is a topic of much focus. In glioma cells, miR-132 suppresses the expression of SIRT1, SREBP1c, and their downstream regulatory genes, reprogramming cholesterol production and adipogenesis. Overexpression of miR-132 can inhibit the proliferation, invasion, migration, and tumorigenicity of cancer cells and induce their apoptosis (Li et al., 2016). miR-449, a potent inducer of apoptosis, cell cycle arrest, and cell differentiation, is under-expressed in various cancers (Chen et al., 2012; Luo et al., 2013; Li et al., 2015b; Li et al., 2015c). miR-449 can inhibit SIRT1-SREBP signaling by reducing the expression of SIRT1, SREBP1c, and its downstream genes *FASN* and *HMGCR*, thereby controlling adipogenesis and cholesterol production in hepatoma cells. Restoration of miR-449 leads to liver tumorigenesis (Zhang et al., 2014). miRNA-128–2 (associated with apoptosis and cholesterol homeostasis) in HepG2, MCF7, and HEK293T cancer cell lines increases SREBP2 expression and decreases SREBP1 expression independent of SIRT1 status (Adlakha et al., 2013). In addition to the SIRT1-SREBP signaling pathway, microRNAs can also downregulate SREBPs in cancer cells through the following signaling pathways. miR-185 and miR-342 control lipogenesis and cholesterol synthesis in prostate cancer cells by inhibiting SREBP1 and SREBP2 expression and downregulating their target genes *FASN* and *HMGCR*. Upregulation of miR-185 and -342 induces caspase-dependent apoptosis in prostate cancer cells and regression of prostate tumors (Li et al., 2013). As one of the earliest discovered mammalian miRNAs, miR-21 is an oncogene in prostate cancer, and its expression level is associated with chemotherapy-resistant castration-resistant prostate cancer (Volinia et al., 2006; Si et al., 2007; Krichevsky and Gabriely, 2009; Wang et al., 2013). miR-21 acts as an oncogene during PCa progression by activating the IRS1/SREBP1 signaling pathway; knockdown of miR-21 can reduce IRS1/SREBP1 in mouse embryonic fibroblasts, mouse prostate tissue, and human PCa cells. Downregulated IRS1-SREBP1 signaling pathway inhibits its downstream targets, such as FASN and ACC, and inhibits prostate cancer progression (Kanagasabai et al., 2022). Long-term exposure to cisplatin develops chemoresistance, desensitizes non-small cell lung carcinoma (NSCLC) cells, and enhances SREBP1-mediated adipogenesis, affecting cancer prognosis. miR-497 induces cisplatin sensitivity in NSCLC cells *via* the SREBP-1/miR-497/SCAP/FASN signaling pathway (Tiong et al., 2022). miR-384 downregulates the oncogene pleiotrophin (PTN) in liver cancer cells by directly binding to 3′-UTR, whereas PTN, an oncogene, acts on liver cancer cells and promotes cell proliferation and adipogenesis through the function of the N-syndecan growth factor. N-syndecan promotes *de novo* lipogenesis in hepatoma cells through the PI3K/Akt/mTORC1/SREBP1c signaling pathway. In hepatocellular carcinoma, HBx inhibits miR-384, upregulating PTN and promoting the proliferation, metastasis, and adipogenesis of cancer cells (Bai et al., 2017). Finally, miR-33a not only cooperates with the SREBP2 cholesterol transcription factor to increase intracellular cholesterol levels (Gerin et al., 2010; Horie et al., 2010; Marquart et al., 2010; Najafi-Shoushtari et al., 2010; Rayner et al., 2010) but also works with miR-33b and their SREBP host gene products to regulate intracellular fatty acid and lipid levels (Gerin et al., 2010; Dávalos et al., 2011). Dysregulation of miR-33a levels may promote tumorigenesis by affecting cholesterol levels. Most studies have shown that miR-33a acts as a tumor suppressor in various cancer cells, inhibiting the proliferation and metastasis of cancer cells (Kuo et al., 2013; Zhang et al., 2015; Han et al., 2016; Karatas et al., 2017; Shan et al., 2017). However, whether miR-33a and miR-33b have extensive cooperation with SREBPs and specific mechanisms in the control of cholesterol and lipid homeostasis during the occurrence and development of cancer still needs further research. We found that most of the current studies on microRNAs and SREBPs are stuck on the effect of lipid accumulation in cancer cells. However, it is still not entirely clear whether the lipid accumulation induced by microRNAs through SREBPs has a direct link to cancer cell phenotype.

In addition to miRNAs, long noncoding RNAs (lncRNAs) also play an irreplaceable role, although the role of regulating SREBPs in cancer is less studied. Hypoxia, a frequent occurrence in solid tumors, is considered an adverse factor for patient prognosis (Vaupel and Mayer, 2007; Bertout et al., 2008). Hypoxia promotes the expression of unknown lncRNAs at the EFNA3 locus through hypoxia-inducible factor (HIF), leading to Ephrin-A3 protein accumulation. Ephrin-A3 expression leads to poor prognosis and increased risk of metastasis in patients with breast cancer (Gómez-Maldonado et al., 2015). Interestingly, HIF-1a directly upregulated EFNA3 expression and Ephrin-A3 accumulation under hypoxic conditions in HCC, similar to the above studies. The authors extended the role of Ephrin-A3 in metabolic reprogramming in a hypoxic microenvironment, reporting for the first time that the Ephrin-A3/Eph receptor A2 (EphA2) axis-promoted SREBP1 maturation and SREBP1-ACLY-mediated metabolic reprogramming are its important downstream signals (Husain et al., 2022). LncRNA metastasis-associated lung adenocarcinoma transcript 1 (MALAT1) is upregulated in many cancers (Gutschner et al., 2013; Hu et al., 2015; Goyal et al., 2021) and involved in the regulation of pre-mRNA splicing (Tripathi et al., 2010; Engreitz et al., 2014). In HCC cells, MALAT1 regulates the expression of genes involved in lipid metabolism, including *SREBF1* and *SCD*, through RNA splicing or transcription (Wang et al., 2021a).

## 5 Treatment in cancer

Given the important regulatory status of SREBPs in lipid metabolism and cancer growth, SREBPs have become potential targets, and the prevention and treatment of cancer can be achieved through small molecules or natural products. There are currently three main treatment strategies: 1) Targeting the translocation of SREBPs from the ER to the Golgi and the cleavage in the Golgi, intervening in the activation of SREBP1/SREBP2; 2) small molecules and natural substances targeting SREBP1 only; and 3) mevalonate signaling pathway inhibition targeting SREBP2 only.

### 5.1 Intervention in the activation of SREBP1/SREBP2

Fatostatin, a non-sterol diarylthiazole derivative, is a specific inhibitor of SREBP activation (Table 1). The important basis for its tumor suppressor effect is to bind to SCAP to inhibit the translocation of SREBP1/SREBP2 from the ER to the Golgi (Kamisuki et al., 2009; Li et al., 2014b; Shao et al., 2016). In prostate cancer, fatostatin inhibits cell proliferation, invasion, and migration of androgen-responsive or insensitive cancer cells. Fatostatin can also induce G2-M cell cycle arrest and induce apoptosis (Li et al., 2014b). Fatostatin inhibits prostate tumor growth and distant lymph node metastasis in mice by inhibiting the activation of SREBPs (Chen et al., 2018). Moreover, the combined use of fatostatin and docetaxel can inhibit the proliferation and apoptosis of prostate cancer cells (especially p53 mutants) to a greater extent than monotherapy (Li et al., 2015d). In endometrial cancer, fatostatin exhibits antitumor effects by inhibiting the SREBPs-regulated metabolic signaling pathways and inducing caspase-mediated apoptosis (Gao et al., 2018). Fatostatin inhibits endometrial cancer cell growth, proliferation, cell cycle, and apoptosis *in vitro* and has antitumor activity *in vivo* (Yao et al., 2020). In breast cancer, fatostatin inhibits cell cycle arrest and apoptosis, especially in estrogen receptor-positive cells. Interestingly, instead of inhibiting lipogenesis by inhibiting the activity of SREBPs, fatostatin caused lipid accumulation through ER stress (Brovkovych et al., 2018). In a variety of cancers, fatostatin inhibits cancer cell proliferation by inhibiting tubulin polymerization and mitosis in cancer cells and disrupting its mitotic microtubule spindle assembly (Gholkar et al., 2016). Betulin, a natural triterpenoid, specifically inhibits the maturation of SREBPs by enhancing the interaction of SCAP with INSIG (Tang et al., 2011). In hepatocellular carcinoma, betulin reduces the level of pro-inflammatory lipids and suppresses inflammation and ER stress by inhibiting the SREBPs signaling pathway, ultimately inhibiting the progression of liver cancer (Li et al., 2017). Betulin inhibits glucose metabolism in hepatocellular carcinoma and enhances the antitumor effect of sorafenib by inhibiting SREBP1 (Yin et al., 2019). In addition to inhibiting the translocation of SCAP/SREBP, inhibiting the Golgi cleavage of SREBPs is also a major therapeutic strategy to prevent their activation. Two important SREBP cleaving enzymes S1P and S2P in the Golgi can be inhibited by PF-429242 and nelfinavir, respectively. In renal cell carcinoma, PF-429242 potently inhibits cell proliferation, migration, and invasion and activates apoptosis by targeting S1P (Wang et al., 2021b). In hepatocellular carcinoma, PF-429242 inhibits viral assembly in infected cells by reducing LD formation, thereby blocking HCV establishment of infection in hepatoma cells (Blanchet et al., 2012; Olmstead et al., 2012). PF-429242 can also synergize with GSK_343_ (EZH2 inhibitor) in hepatocellular carcinoma, enhancing the anticancer activity of GSK_343_ (Yang et al., 2019). In glioblastoma, PF-429242 downregulates steroid, isoprenoid, and unsaturated fatty acid biosynthetic signaling pathways and upregulates pro-inflammatory genes to reduce cancer cell viability and promote apoptosis (Caruana et al., 2017). In liposarcoma, nelfinavir upregulates the precursors SREBP1 and ATF6 by inhibiting the cleavage of S2P, resulting in ER stress and induction of apoptosis (Guan et al., 2011). In prostate cancer, nelfinavir also reduces the proliferation of castration-resistant prostate cancer and promotes apoptosis through the accumulation of unprocessed SREBP1 and ATF6 (Guan et al., 2012; Guan et al., 2015). Notably, in these three nelfinavir reports, the authors used another S2P-specific inhibitor, 1,10-phenanthroline, and achieved similar effects to nelfinavir in cancer.

**TABLE 1:** Treatment in cancer.

Targets	Drugs	Cancer types	Mechanisms
SREBP1/SREBP2	Fatostatin	Prostate cancer	Inhibits cell proliferation, invasion, and migration of cancer cells and causes G2-M cell cycle arrest and induces apoptosis (Li et al., 2014b)
			Inhibits prostate tumor growth and distant lymph node metastasis in mice by inhibiting the activation of SREBPs (Chen et al., 2018)
			Combined use with docetaxel can inhibit the proliferation and apoptosis of prostate cancer cells (especially p53 mutants) to a greater extent (Li et al., 2015d)
		Endometrial cancer	Inhibits metabolic signaling pathways regulated by SREBPs and induces caspase-mediated apoptosis (Gao et al., 2018)
			Inhibits growth, proliferation, cell cycle, and apoptosis and has antitumor activity *in vivo* (Yao et al., 2020)
		Breast cancer	Causes cell cycle arrest and apoptosis by ER stress (Brovkovych et al., 2018)
		Multiple cancers	Inhibits tubulin polymerization and mitosis and perturbs its mitotic microtubule spindle assembly (Gholkar et al., 2016)
	Betulin	Hepatocellular carcinoma	Reduces the levels of pro-inflammatory lipids and suppresses inflammation and ER stress by inhibiting the translocation of SREBPs (Li et al., 2017)
			Inhibits glucose metabolism and enhances the antitumor effect of Sorafenib by inhibiting SREBP1 (Yin et al., 2019)
	PF-429242	Renal cell carcinoma	Inhibits cell proliferation, migration, and invasion, and activates apoptosis by targeting S1P (Wang et al., 2021b)
		Hepatocellular carcinoma	Inhibits viral assembly in infected cells by reducing LD formation, thereby blocking HCV establishment of infection (Blanchet et al., 2012; Olmstead et al., 2012)
			Synergizes with GSK_343_, enhancing the anticancer activity of GSK_343_ (Yang et al., 2019)
		Glioblastoma	Downregulates steroid, isoprenoid, and unsaturated fatty acid biosynthetic signaling pathways and upregulates pro-inflammatory genes to reduce cancer cell viability and promote apoptosis (Caruana et al., 2017)
	Nelfinavir,1,10-phenanthroline	Liposarcoma	Upregulates the precursors SREBP1 and ATF6 by inhibiting the cleavage of S2P, resulting in ER stress and induction of apoptosis (Guan et al., 2011)
		Prostate cancer	Reduces the proliferation of castration-resistant prostate cancer and promotes apoptosis through the accumulation of unprocessed SREBP1 and ATF6 (Guan et al., 2012; Guan et al., 2015)

### 5.2 Small molecules and natural substances targeting SREBP1

Several classes of small molecules or new formulations have been reported as modulators of adipogenesis targeting SREBP1 in cancer (Table 2). Apatinib, an inhibitor of VEGFR2, downregulates GPX4 expression by inhibiting SREBP1a, thereby inducing lipid peroxidation and ferroptosis in gastric cancer (Zhao et al., 2021). Mollugin, with anti-inflammatory and apoptotic effects, inhibits proliferation and induces apoptosis in HER2-overexpressing breast and ovarian cancers by regulating SREBP1c and its target gene *FASN* through the HER2/Akt signaling pathway (Do et al., 2013a). WY 14,643 and troglitazone, agonists of PPARα and PPARγ, respectively, inhibit SREBP1 activation through the upregulation of INSIG, ultimately reducing triacylglycerol synthesis in hepatoma cells (König et al., 2009). Azathioprine, an immunosuppressant, inhibits elevated lipid metabolism *via* the EGFR/AKT/SREBP1 signaling pathway and induces ER stress to induce apoptosis in glioblastoma cells (Nam et al., 2021). GW3965, a hepatic X receptor agonist, promotes glioblastoma cell death by inhibiting the EGFR/AKT/SREBP1/LDLR signaling pathway (Guo et al., 2011). Gefitinib induces downregulation of SREBP1 in non-small cell lung cancer treatment-sensitive cells, inhibits fatty acid synthesis, and alters the ratio of saturated to unsaturated phospholipids (Xu et al., 2021a). Metformin inhibits bladder cancer cell growth by controlling lipid synthesis *via* the Clusterin/SREBP1c/FASN axis (Deng et al., 2021). Proxalutamide, an AR antagonist, significantly inhibits prostate cancer cell proliferation and migration and induces apoptosis. Proxalutamide also inhibits the expression of ACL, ACC, FASN, and SREBP1 to reduce lipid droplet levels and triglyceride content in cancer cells (Gu et al., 2021). ASC-J9, as an AR degradation enhancer, inhibits the proliferation and invasion of prostate cancer cells through the AR/SREBP1/FASN and PI3K/Akt/SREBP1/FASN signaling pathways according to whether AR is positive or not (Wen et al., 2016).

**TABLE 2:** Treatment in cancer.

Targets	Drugs	Cancer types	Mechanisms
SREBP1	Apatinib	Gastric cancer	Downregulates GPX4 expression by inhibiting SREBP1a, thereby inducing lipid peroxidation and ferroptosis (Zhao et al., 2021)
	Mollugin	Breast/ovarian cancers	Inhibits proliferation and induces apoptosis through the HER2/Akt/SREBP1c/FASN signaling pathway (Do et al., 2013a)
	WY 14,643 and troglitazone	Hepatocellular carcinoma	inhibit SREBP1 activation through the upregulation of INSIG, ultimately reducing triacylglycerol synthesis (König et al., 2009)
	Azathioprine	Glioblastoma	Inhibits elevated lipid metabolism *via* EGFR/AKT/SREBP1 signaling pathway and induces ER stress to induce apoptosis (Nam et al., 2021)
	GW3965	Glioblastoma	Promotes cell death by inhibiting the EGFR/AKT/SREBP1/LDLR signaling pathway (Guo et al., 2011)
	Gefitinib	Non-small cell lung cancer	Induces downregulation of SREBP1, and alters the ratio of saturated to unsaturated phospholipids (Xu et al., 2021a)
	Metformin	Bladder cancer	Inhibits cancer cell growth by controlling lipid synthesis *via* the Clusterin/SREBP1c/FASN axis (Deng et al., 2021)
	Proxalutamide	Prostate cancer	Inhibits proliferation, migration, and expression of ACL, ACC, FASN, and SREBP1 to reduce lipid droplet levels and triglyceride content (Gu et al., 2021)
	ASC-J9	Prostate cancer	Inhibits the proliferation and invasion of prostate cancer cells by the AR/SREBP1/FASN or API3K/Akt/SREBP1/FASN signaling pathways according to whether AR is positive or not (Wen et al., 2016)
	RA-XII	Colorectal cancer	Inhibits the growth and metastasis of colorectal tumors by reducing the expression of SREBP1 and its target genes (Wang et al., 2019)
	Berberin	Colon cancer	Mediates lipogenesis by inhibiting SCAP expression and SREBP1 activation, thereby inhibiting cell proliferation and colon cancer xenograft growth (Liu et al., 2020)
	Ginsenoside Rh2	Non-small cell lung cancer	Reverses cyclophosphamide-induced immunodeficiency by inhibiting the expression of SREBP1 and affecting the interaction of SREBP1 with FASN (Qian et al., 2019)
	Davallia formosana ethanol extract	Prostate cancer	Inhibits proliferation, migration, and invasion by inhibiting the expression of SREBP1 and FASN and reducing the expression of AR and PSA (Hsieh et al., 2020)
	Curcumin	Hepatocarcinoma	Reduces adipogenesis by activating the phosphorylation of AMPK to reduce the expression of SREBP1 and FASN and increases the expression of PPARα (Kang et al., 2013)
	Oleiferasaponin A₂	Hepatocarcinoma	Inhibits lipid accumulation by significantly down-regulating fatty acid synthesis genes and up-regulating fatty acid β-oxidation genes (Di et al., 2018)
	Piperine	Breast cancer	Reduces the expression of SREBP1 and FASN by inhibiting ERK1/2 signaling, and also inhibits the proliferation by activating caspase-3 and cleaving PARP (Do et al., 2013b)

Notably, natural substances can also modulate SREBP1 for the treatment of different cancers. The natural cyclic peptide RA-XII, isolated from Rubia yunnanensis, inhibits the growth and metastasis of colorectal tumors by reducing the expression of SREBP1 and its target genes (Wang et al., 2019). Berberin, extracted from the Rizoma coptidis, mediates lipogenesis by inhibiting SCAP expression and SREBP1 activation, thereby inhibiting colon cancer cell proliferation and colon cancer xenograft growth (Liu et al., 2020). Ginsenoside Rh2, an extract from ginseng, reverses cyclophosphamide-induced immunodeficiency in non-small cell lung cancer by inhibiting the expression of SREBP1 and its nuclear translocation and affecting the interaction of SREBP1 with FASN (Qian et al., 2019). Davallia formosana ethanol extract inhibits proliferation, migration, and invasion in prostate cancer cells by inhibiting the expression of SREBP1 and FASN and reducing the expression of AR and prostate-specific antigen (PSA) (Hsieh et al., 2020). Curcumin, the yellow pigment from turmeric, exhibits anti-cancer and antioxidant effects, especially in hepatocarcinoma. Curcumin can not only reduce adipogenesis in hepatoma cells by activating the phosphorylation of AMPK to reduce SREBP1 and the expression of FASN but also increase the expression of PPARα and increase its antioxidant effect (Kang et al., 2013). Oleiferasaponin A₂, isolated from the defatted seeds of Camellia oleifera, inhibits lipid accumulation in hepatoma cells by significantly down-regulating fatty acid synthesis genes (the genes encoding SREBP1c, FASN, ACC) and up-regulating fatty acid β-oxidation genes (the genes encoding PPARα, ACOX-1, CPT-1) (Di et al., 2018). Piperine, extracted from black pepper, significantly reduces the expression of SREBP1 and FASN by inhibiting ERK1/2 signaling and also inhibits the proliferation of HER2-overexpressing breast cancer cells by activating caspase-3 and cleaving PARP (Do et al., 2013b). Interestingly, physical exercise induces changes in lipid metabolism signaling pathways (decreased expression of CD36, SREBP1, and SCAP) and prostate cell apoptosis, suggesting that physical exercise may be a new therapeutic strategy for the treatment of prostate cancer (Teixeira et al., 2020).

### 5.3 Mevalonate signaling pathway inhibition targeting SREBP2

Cholesterol metabolism, a risk signal and driver of tumor growth, is controlled by SREBP2 over the expression of important cholesterol biosynthetic genes and is associated with prognosis in multiple cancers (Table 3). HMGCR (the rate-limiting enzyme for cholesterol synthesis) is a key target for inhibiting the SREBP2 signaling pathway for cancer therapy. Statins, the most classic HMGCR inhibitors, have become the cornerstone of therapy in cancer patients with high cholesterol levels and have also reduced cancer incidence and recurrence (Khurana et al., 2007; Singh et al., 2009; Tran et al., 2020). Statins are mainly divided into two types, fungal fermentation or chemical synthesis, including type 1, lovastatin, mevastatin, and simvastatin, and type 2, fluvastatin and atorvastatin (Xue et al., 2020). In breast cancer, atorvastatin, lovastatin, and simvastatin alter the expression of 50 genes with a shared cluster of 37 genes, including the Hippo, Notch, and Wnt signaling pathways, preventing the EMT process (Koohestanimobarhan et al., 2019). Lovastatin can signal through PPARγ (a breast cancer-associated tumor suppressor) and upregulate *PTEN* at the transcriptional level (Teresi et al., 2006). Simvastatin further contributes to breast cancer cell death by inducing the inactivation of PI3K/Akt and MAPK/Erk signaling (Wang et al., 2016). In breast cancer cells, elevated levels of mevalonate produced by SREBP2 transcriptional activity promote the activation of YAP/TAZ signaling, whereas cerivastatin inhibits this signaling pathway and hinders the nuclear localization and transcription of YAP/TAZ (Piccolo et al., 2014; Sorrentino et al., 2014). Lovastatin reduces the expression of CYR61 *via* SREBP2/miR-33a, which in turn inhibits osteosarcoma cell invasion (Huang et al., 2018). In thymic carcinoma, Fluvastatin inhibits HMGCR to suppresses cell proliferation, which might be mediated by inhibiting the production of geranylgeranyl-pyrophosphate (Hayashi et al., 2020). Fluvastatin alters Braf/MEK/ERK1/2 and Akt signaling pathways by inhibiting HMGCR, which can inhibit cell growth, induce apoptosis, and inhibit tumorigenesis in non-small cell lung cancer (Zhang et al., 2019). In colorectal cancer, simvastatin suppresses PD-L1 expression and promotes antitumor immunity by inhibiting lncRNA SNHG29 expression and its mediated YAP activation (Ni et al., 2021). Simvastatin and fluvastatin reduce cell proliferation and induce apoptosis mediated by phosphorylation downregulation of the AKT/FOXO1 signaling pathway in prostate cancer cells (Deng et al., 2019). In potential-resistant prostate cancer, reduction of AR by simvastatin *via* inhibition of the mTOR signaling pathway overcomes enzalutamide resistance (Kong et al., 2018). In addition to regulating multiple signaling pathways to affect tumors, statins can also synergize with multiple drugs. Simvastatin and tricostatin A, an HDAC inhibitor, inhibit invasion and growth and exhibit synergistic antitumor effects in ORP5-expressing pancreatic cancer cells. Combination therapy can inhibit the growth of cancer cells to a greater extent (Ishikawa et al., 2010). Archazolid B, a vacuolar H (+)-ATPase inhibitor, causes dramatic disturbance of cholesterol homeostasis, activation of SREBP2 and upregulation of the target gene *HMGCR*. The combination of archazolid B and fluvastatin affects cholesterol biosynthesis to enhance archazolid B-induced cell death (Hamm et al., 2014). Dipyridamole, a phosphodiesterase inhibitor, can retain the SCAP-SREBP complex in the ER by stabilizing the INSIG protein when acting alone, and when combined with atorvastatin can further enhance the inhibition of cervical cancer cell growth by atorvastatin (Esquejo et al., 2021). Dipyridamole inhibits fluvastatin-induced SREBP2 activation and enhances apoptosis in statin-insensitive prostate cancer cells (Longo et al., 2019). Combination treatment of 25-hydroxycholesterol with simvastatin significantly enhances statin cytotoxicity and antitumor activity in ovarian cancer cells (Casella et al., 2014). In Oral and Esophageal Cancer, pitavastatin suppresses AKT and ERK signaling in cells to inhibit tumor growth alone. Importantly, pitavastatin significantly reduces tumor growth in cooperation with capmatinib, a MET-specific inhibitor (Xu et al., 2021b). Simvastatin enhances the proapoptotic activity of venetoclax (B cell lymphoma-2 inhibitor) in primary leukemia and lymphoma cells but not normal peripheral blood mononuclear cells (Lee et al., 2018). Interestingly, Low serum cholesterol levels are positively associated with poorer survival outcomes in patients with renal cell carcinoma. Lovastatin fails to inhibit tumor progression, but instead increases glycolysis levels through regulated HSP90 expression levels, leading to lactate accumulation and acceleration of renal cell carcinoma development. However, Shikonin (a PKM2 inhibitor) can reverse the tumor-promoting effect of lovastatin (Huang et al., 2021).

**TABLE 3:** Treatment in cancer.

Targets	Drugs	Cancer types	Mechanisms
SREBP2	AtorvastatinL	Breast cancer	Alter the expression of 50 genes with a shared cluster of 37 genes, including the Hippo, Notch, and Wnt signaling pathways, preventing the EMT process (Koohestanimobarhan et al., 2019)
	Ovastatin		
	Simvastatin		
	Lovastatin	Breast cancer	Signal through PPARγ and upregulate *PTEN* at the transcriptional level (Teresi et al., 2006)
	Simvastatin	Breast cancer	Contributes to breast cancer cell death by inducing inactivation of PI3K/Akt and MAPK/Erk signaling (Wang et al., 2016)
	Cerivastatin	Breast cancer	Inhibits the elevated levels of mevalonate produced by the transcriptional activity of SREBP2 and impedes the nuclear localization and transcription of YAP/TAZ (Piccolo et al., 2014; Sorrentino et al., 2014)
	Lovastatin	Osteosarcoma	Reduces the expression of CYR61 *via* SREBP2/miR-33a, which in turn inhibits osteosarcoma cell invasion (Huang et al., 2018)
	Fluvastatin	Thymic carcinoma	Inhibits HMGCR to suppresses cell proliferation, which might be mediated by inhibiting the production of geranylgeranyl-pyrophosphate (Hayashi et al., 2020)
	Fluvastatin	Non-small cell lung cancer	Alters Braf/MEK/ERK1/2 and Akt signaling pathways by inhibiting HMGCR, which can inhibit cell growth, induce apoptosis, and inhibit tumorigenesis in non-small cell lung cancer (Zhang et al., 2019)
	Simvastatin	Colorectal cancer	Suppresses PD-L1 expression and promotes antitumor immunity by inhibiting lncRNA SNHG29 expression and its mediated YAP activation (Ni et al., 2021)
	Simvastatin	Prostate cancer	Reduce cell proliferation and induce apoptosis mediated by phosphorylation downregulation of AKT/FOXO1 signaling pathway (Deng et al., 2019)
	Fluvastatin		
	Simvastatin	Prostate cancer	Overcome enzalutamide resistance by reducing AR by inhibiting the mTOR signaling pathway (Kong et al., 2018)
	Simvastatin	Pancreatic cancer	Inhibit invasion and growth and exhibit synergistic antitumor effects in ORP5-expressing pancreatic cancer cells (Ishikawa et al., 2010)
	Tricostatin A		
	Fluvastatin	Bladder cancer	Affect cholesterol biosynthesis to enhance archazolid B-induced cell killing (Hamm et al., 2014)
	Archazolid B		
	Atorvastatin	Cervical cancer	Retain the SCAP-SREBP complex in the ER by stabilizing the INSIG protein (Esquejo et al., 2021)
	Dipyridamole		
	Fluvastatin	Prostate cancer	Inhibit SREBP2 activation and promotes apoptosis in statin-insensitive prostate cancer cells (Longo et al., 2019)
	Dipyridamole		
	Simvastatin	Ovarian cancer	Significantly enhance the cytotoxicity and antitumor activity of statins against ovarian cancer cells (Casella et al., 2014)
	25-hydroxycholesterol		
	Pitavastatin	Oral and Esophageal Cancer	Suppresses AKT and ERK signaling to inhibit tumor growth alone, and significantly reduces tumor growth in cooperation with capmatinib (Xu et al., 2021b)
	Capmatinib		
	Simvastatin	Primary leukemia and lymphoma	Enhances the proapoptotic activity of venetoclax (B cell lymphoma-2 inhibitor) in primary leukemia and lymphoma cells but not normal peripheral blood mononuclear cells (Lee et al., 2018)
	Venetoclax		
	Lovastatin	Renal cell carcinoma	Increases glycolysis levels through regulated HSP90 expression levels, leading to lactate accumulation and acceleration of renal cell carcinoma development. The tumor-promoting effect of lovastatin is reversed by Shikonin (Huang et al., 2021)
	Shikonin		

## 6 Conclusion

In conclusion, with the reprogramming of lipid metabolism as an emerging hallmark of cancer, we need to deepen our understanding of the dysregulation of lipid metabolism in cancer. Intracellular oncogenic signal transduction, DNA, RNA, cytokines, growth factors, and tumor microenvironment can all regulate lipid metabolism in tumor cells. Aberrant lipid metabolism can also influence oncogenic signaling pathways in cancer cells. SREBPs, as core transcription factors in lipid metabolism, link oncogenic signal transduction with changes in lipid metabolism and play an important role in malignant tumors. Tumor cells voraciously upregulate SREBPs through different subcellular localizations, including the ER, Golgi, and nucleus, thereby further regulating the lipid uptake, lipid production (FAs and cholesterol), and lipid decomposition of tumor cells, serving the tumor cells themselves. In particular, numerous signaling molecules can regulate the transcription, expression, activation, stability, and binding of SREBPs, which can mediate downstream signaling pathways, leading to tumor proliferation, invasion, metastasis, apoptosis, epithelial-mesenchymal transition, and ER stress. An in-depth study of the specific regulatory mechanisms of SREBPs in tumors will provide new and exciting therapeutic opportunities to eliminate cancer with the best efficacy and minimal side effects.

## Abbreviations

SREBPs, Sterol regulatory element binding proteins ER, endoplasmic reticulum SCAP, SREBP cleavage-activating protein INSIG, insulin-induced gene protein COPII, the Coat Protein complex II S1P, site 1 proteases S2P, site 2 proteases FAs, fatty acids FASN, fatty acid synthase ACC1, acetyl-CoA carboxylase 1 HCC, hepatocellular carcinoma 25-HC, 25-hydroxycholesterol SCD, stearoyl-CoA desaturase HCV, hepatitis C Virus ABCA1, ATP-binding cassette transporter A1 SOAT, sterol o-acyltransferase EPA, Eicosapentaenoic acid DHA, docosahexaenoic acid FFA4, free fatty acid receptor 4 LDLR, low-density lipoprotein receptor PKM2, pyruvate kinase M2 PTPRO, protein tyrosine phosphatase receptor type O YAP, Yes-associated protein AMPK, AMP-activated protein kinase PD-L1, programmed death 1 ligand 1 EMT, epithelial-mesenchymal transition HBXIP, Hepatitis B X-interacting protein LXR, liver X receptor IDH1, isocitrate dehydrogenase 1 FDXR, ferredoxin reductase TMEM33, ER transmembrane protein 33 SPRING/C12ORF49, SREBP-regulated gene DHT, dihydrotestosterone GP73, Golgi Protein 73 PCK1, phosphorylate cytosolic phosphoenolpyruvate carboxykinase 1 LPCAT1, lysophosphatidylcholine acyltransferase 1 LDs, lipid droplets HSP90, heat shock protein 90 PAQR3, progesterone and fat receptor 3 PPARγ, peroxisome-proliferator-activated receptor-gamma CREB, cAMP-response element binding protein RANKL, receptor activators for nuclear factor-κB ligand miRNAs, small non-coding RNAs lncRNAs, long noncoding RNAs PTN, pleiotrophin HIF, hypoxia-inducible factor EphA2, Ephrin-A3/Eph receptor A2 MALAT1, metastasis-associated lung adenocarcinoma transcript 1.
